# Wear and roughness analysis of two highly filled flowable composites

**DOI:** 10.1007/s10266-024-01013-0

**Published:** 2024-10-07

**Authors:** Vittorio Checchi, Luigi Generali, Laura Corciolani, Lorenzo Breschi, Claudia Mazzitelli, Tatjana Maravic

**Affiliations:** 1 Department of Surgery, Medicine, Dentistry and Morphological Sciences With Transplant Surgery, Oncology and Regenerative Medicine Relevance, University of Modena and Reggio Emilia, Modena, Italy; 2 Department of Biomedical and Neuromotor Sciences, University of Bologna, Bologna, Italy

**Keywords:** Resin composite, Flowable composite, Light curing, Surface roughness, Wear

## Abstract

This in vitro study aimed to evaluate surface roughness and wear of highly filled flowables and traditional packable composites. Additionally, the effect of polymerization time on these parameters was evaluated. Two flowable higly filled composites (CMf—Clearfil Majesty ES flow-low viscosity, Kuraray and GUf—Gaenial Universal Injectable, GC) and two packable composites (CM—Clearfil Majesty ES-2, Kuraray and GU—Gaenial A'CHORD, GC) were used to create 160 specimens (*n* = 40;8 × 6 × 4mm). For each tested material, two subgroups were considered according to the polymerization time (*n* = 20): 10 s or 80 s. After setting, the specimens were subjected to chewing simulations (240.000 cycles, 20N), and wear was measured by the laser integrated in the chewing simulator. The surface roughness was measured using a rugosimeter, before and after chewing cycles. Two representative specimens per group were observed under scanning electron microscope (SEM). Data were collected and statistically analyzed (*p* < 0.05). Wear analysis highlighted statistically significant differences between the groups: CMf10-CMf80 (*p* = 0.000), CMf10-CM10 (*p* = 0.019), CMf10-GUf10 (*p* = 0.002), CM10-CM80 (*p* = 0.000), CM80-GUf80 (*p* = 0.02), GUf10-GUf80 (*p* = 0.000), GUf10-GU10 (*p* = 0.043) and GU10-GU80 (*p* = 0.013). Statistically significant differences in surface roughness were highlighted between the groups: CMf10-CMf80 (*p* = 0.038), CMf80-CM80 (*p* = 0.019), CMf80-GU80 (*p* = 0.010), CM80-GUf80 (*p* = 0.34) and GUf80-GU80 (*p* = 0.003). Surface roughness and wear of highly filled flowable composites were comparable to that of traditional paste composites. Furthermore, a longer curing time leads to an improvement in the mechanical properties of the composites. Highly filled flowables can be a valid alternative to paste composites in occlusal areas due to its similar surface roughness and wear values, especially when overcured.

## Introduction

Resin composite posterior teeth restorations involving the occlusal portion of the teeth are a common clinical evinience. During chewing activity, these restorations are subjected to continuous stresses that can undermine their survival in the oral cavity over time due to wear, fracture or increased roughness that may lead to bacterial accumulation and increase the risk of secondary caries [1, 2].

The physical–mechanical characteristics of resin composite materials are largely dictated by the filler loading and size [3–5]. Traditionally, the filler content has allowed to distinguish between bulk conventional composites and flowable composites [6–8]. The smaller filler load of flowable composites confers a fluid property and enhanced polishability to the material, making them an optimal clinical choice when used as liners, sealants or class V restorations [9]. In contrast, higher wear rate and inferior mechanical properties have been demonstrated when flowable resins have been compared to conventional composite [10]*.*

Nevertheless, the use of flowable composites under occlusal loading has recently been increasingly exploited, favored by the advent of the injection technique and the introduction of newer restorative approaches [11, 12]. In light of the even higher possibilities in restorative dentistry, impressive efforts have been invested toward improving the mechanical properties of flowable composites [13]. In particular, the incorporation of a high-volume fraction of filler particles to increase wear resistance, reduce surface degradation and provide long-lasting restoration is one of the most followed topics in modern materials’ technology [9, 14].

Another important factor influencing the mechanical properties and durability of resin composites is the curing efficacy: in other words the amount of resin that can be converted from monomers to polymer [15]. Inadequate polymerization is significantly associated with a decrease in the physical properties of resin composites [16], whereas a more efficient polymerization increases resistance to wear and fracture and improves the color stability of the composite resin [17, 18]. To the best of the authors’ knowledge, the information available in the scientific literature on the effects of polymerization on surface characteristics and wear reasistance of the recently introduced highly filled flowable composite is scarce.

Therefore, the aim of ths in vitro study was to compare the surface roughness and wear resistance of highly filled flowable and conventional composites before and after chewing simulation when submitted to different curing times. Particularly, the null hypotheses were that: 1) no differences in surface roughness exist between the two materials; 2) the two composite materials exhibit similar wear resistance; and 3) curing time does not influence the surface roughness and wear resistance of the tested materials.

## Methods

To calculate the sample size, a pilot study (5 samples per group) was used and the means and standard deviation were inserted in G*Power 3.1 for Mac. According to the calculated effect size f = 0,3,812,953, α error probability = 0.05 and power (1-β error probability) = 0.95, the required sample size for roughness analysis would be 13 per group. As for wear analyses, the calculated effect size f = 1,077033, α error probability = 0.05 and power (1-β error probability) = 0.95 required a sample size of four specimens per group. However, to reaffirm our results, an elevated number of specimens per group (*n* = 20) was used for both analyses, so as to reach the commonly reported sample size suggested for dental materials research.

A silicone mold was used to create 160 (8 × 6 × 4 mm) composite specimens using two highly loaded flowable composites (Clearfil Majesty ES flow—low viscosity—Kuraray Noritake, Tokyo, Japan; Gaenial Universal Injectable–GC, Hongo, Bunkyoku, Tokyo) and two traditional composite pastes (Clearfil Majesty ES-2–Kuraray; Gaenial A'CHORD–GC) (*n* = 40 per group). Each group was then divided into two subgroups according to the curing time (*n* = 20): light curing for 10 s or 80 s. Light curing was performed with a light-emitting diode (LED; Mectron Starlight pro LED lamp, middle-intensity blue LED, 1400 mW/cm2). The groups so formed are schematically presented in Table 1.

**Table 1:** Composites used in this study

Group	Composite	Color	Curing time (s)	LOT number	Fillers	Particle size	Filler volume (vol %)	Filler weight (wt %)
CMf10	Clearfil Majesty ES Flow (Kuraray)	A2	10	5C0037 5C0038	Silanated barium glass, silanated colloidal silica	0.18–3.5µ	64	78
CMf80	Clearfil Majesty ES Flow (Kuraray)	A3	80	5T0056 4L0056	Silanated barium glass, silanated colloidal silica	0.18–3.5µ	64	78
CM10	Clearfil Majesty ES-2 (Kuraray)	A1	10	840,031	Silanated barium glass	0.7µ	66	78
CM80	Clearfil Majesty ES-2 (Kuraray)	A1	80	C90032	Silanated barium glass	0.7µ	66	78
GUf10	Gaenial Universal Injectable (GC)	A2	10	2,110,121	Silicon dioxide, strontium–glass	Silicon dioxide: 0.016µ; strontium–glass: 0.2µ	50	69
GUf80	Gaenial Universal Injectable (GC)	A2	80	2,108,181	Silicon dioxide, strontium–glass	Silicon dioxide: 0.016µ; strontium–glass: 0.2µ	50	69
GU10	Gaenial A’CHORD (GC)	A2	10	2,102,091	Lanthanide, strontium	N.A	41	82
GU80	Gaenial A’CHORD (GC)	A2	80	2,102,251	Lanthanide, strontium	N.A	41	82

In detail, each composite was layered 2 mm at a time. Each layer was cured 10 s, keeping the lamp as close to the surface as possible. Before curing the final layer, a Mylar sheet was placed on the specimens to obtain a smooth surface. The final layer was cured for 10 s or 80 s, depending on which subgroup the sample belonged to. After light curing, the specimens were released from the mold and placed in a laboratory oven at 37 °C for 24 h to finalize the polymerization reaction. All the specimens were then subjected to polishing procedures, using two paper discs with different granulometry sizes (1.200 and 4.000, respectively). Each disc was subjected to a continuous jet of water for 20 s. The specimens were then placed in an ultrasonic distilled water bath for 3 min, dried, and stored in a light-free box.

### Wear measurements

The specimens were fixed in acrylic resin (Technovit 4071, Kulzer, Hanau, Germany) and positioned inside the chambers of the chewing simulator equipped with a wear detector (CS4.4 Mechatronik, Munich, Germany) to be subjected to circular cycles (circle diameter 5.0 mm; intrusion depth 1.0 mm; speed 30.0 mm/s; movement pattern CW; cycle target 240.000; antagonist steatite sphere, diameter 6 mm). The wear values, expressed in mm, were recorded every 2.400 cycles.

### Surface roughness analysis

Surface roughness was measured on each specimen using a rugosimeter (SJ-201, Mitutoyo Surftest, Kawasaki, Japan), before (T_0_) and after being subjected to chewing cycles (T_1_). This instrument has a detector (stylus tip R: 10 μm) that slides over the surface to be analyzed and reports a numerical value expressed in μm (with cutoff length parameter: 0.25 × 5). The detector was randomly positioned on the surface of the sample three times and the surface roughness value recorded in three different areas. The average of these three values constituted the initial roughness value of each sample. After the chewing simulation (T_1_), three other measurements per specimen were taken on the border between the chewed part and the intact surface. The average of these thre measurements constituted the final roughness value of each specimen.

The measurements were recorded in an Excel file and the difference between T_1_ and T_0_ was calculated for each specimen.

### FEG–SEM analysis

After the chewing process, two specimens per group were randomly selected to be analyzed under a scanning electron microscope (SEM-FEG Nova NANOSEM 450 microscope, FEI Company—ThermoFisherScientific, Hillsboro, OR, USA).

The specimens were mounted on an aluminum stub using a graphite-based conductive double-sided tape. They were subsequently coated with a thin layer of pure gold using a metallizer (Emitech K550, Labtech International Ltd, UK).

Four images were acquired for each specimen at different magnifications, from 105 to 1000X, to observe the transition zone between the worn area and the area that had not been masticated.

### Statistical analysis

The associations among the outcome measures and groups were assessed using linear regression models test. Therefore, it was not necessary to evaluate if data presented normal distribution or homoscedasticity. For each outcome, univariable analyses were performed, estimating a model for each couple of groups. The adequacy of these models was verified through a visual analysis of residuals graphs.

Results were considered statistically significant when associated with a p value lower than the alpha level of 0.05 (*p* value < 0.05). Analyses were carried out using a statistical software (R software, version 4.2.2, R Core Team).

To asses the validity of the assumption underlying the estimated linear regression models, the standardized residual distribution and Q–Q plot were graphically examined. In detail, the following graphs were analyzed: check normality, homoskedasticity, normality and influential observations.

## Results

Roughness and wear variables were described using means and standard deviations, and descriptive statistics were reported separately by groups. Results were reported as the mean difference (MD) with 95% confidence intervals and p values.

### Surface roughness

Surface roughness values are reported in Table 2 as mean and standard deviation for each group.

**Table 2:** Initial, final and delta surface roughness values for each group (mean ± std. dev.)

Group	Mean initial roughness value	Mean final roughness value	Differences initial–final (delta)
CMf10	0.27 (± 0.13)	0.66 (± 0.12)	0.4 (± 0.19)
CMf80	0.13 (± 0.05)	0.42 (± 0.10)	0.29 (± 0.10)
CM10	0.50 (± 0.29)	0.79 (± 0.15)	0.29 (± 0.20)
CM80	0.52 (± 0.23)	0.69 (± 0.12)	0.17 (± 0.21)
GUf10	0.25 (± 0.14)	0.60 (± 0.12)	0.36 (± 0.18)
GUf80	0.17 (± 0.07)	0.45 (± 0.09)	0.28 (± 0.09)
GU10	0.47 (± 0.25)	0.80 (± 0.14)	0.33 (± 0.27)
GU80	0.27 (± 0.14)	0.68 (± 0.14)	0.41 (± 0.17)

Roughness values of groups cured for 80 s were lower than the corresponding groups cured for 10 s. Moreover, although the initial and final surface roughness values of the highly loaded flowable composites (groups CMf and GUf) were better than the packable composite counterparts, in the variation (delta) between the initial and final situation flowable composites presented less performing values.

The best initial average roughness values were represented by groups CMf80 (0.13 ± 0.05) and GUf80 (0.17 ± 0.07), whereas the best final average roughness values were also represented by the same groups CMf80 (0.42 ± 0.1) and GUf80 (0.45 ± 0.09).

Regarding the initial roughness values, all comparisons between groups cured for 10 and 80 s showed statistically significant differences (Table 3).

**Table 3:** Comparison of initial roughness values between pairs of groups (* = statistically significant values)

Comparison	MD	95% Conf. Int		*p* value
CMf10 vs CMf80	− 0.14	− 0.20	− 0.08	< 0.001*
CMf10 vs CM10	0.23	0.08	0.37	0.003*
CMf10 vs GUf10	− 0.02	− 0.11	0.06	0.589
CMf10 vs GU10	0.21	0.08	0.33	0.003*
CMf80 vs CM80	0.39	0.29	0.50	< 0.001*
CMf80 vs GUf80	0.04	0.00	0.08	0.046*
CMf80 vs GU80	0.14	0.07	0.21	< 0.001*
CM10 vs CM80	0.03	− 0.14	0.19	0.754
CM10 vs GUf10	− 0.25	− 0.40	− 0.11	0.001*
CM80 vs GUf80	− 0.35	− 0.46	− 0.24	< 0.001*
GUf10 vs GUf80	− 0.08	− 0.15	− 0.01	0.034*
GUf10 vs GU10	0.23	0.10	0.36	0.001*
GUf80 vs GU80	0.10	0.03	0.17	0.007*
GU10 vs GU80	− 0.20	− 0.33	− 0.07	0.004*

Specimens light cured for 80 s presented lower roughness values, except for the CM group in which both values were comparable and no statistical differences were shown (*p* = 0.754). Moreover, the group CMf80 showed the best (lowest) initial roughness values, followed by group GUf80. All comparisons of initial roughness values between flowable and packable composites gave statistically significant differences (*p* < 0.05), and all packable resins showed initial roughness values higher than flowables. The only comparison that did not produce significant differences was between groups CMf10 and GUf10 (*p* = 0.589).

Also regarding the final roughness values, the trend seems to remain the same (Table 4).

**Table 4:** Comparison of final roughness values between pairs of groups (* = statistically significant values)

Comparison	MD	95% Conf. Int		*p* value
CMf10 vs CMf80	− 0.24	− 0.31	− 0.17	< 0.001*
CMf10 vs CM10	0.13	0.04	0.21	0.005*
CMf10 vs GUf10	− 0.06	− 0.14	0.01	0.106
CMf10 vs GU10	0.14	0.05	0.22	0.002*
CMf80 vs CM80	0.27	0.20	0.34	< 0.001*
CMf80 vs GUf80	0.03	− 0.04	0.09	0.403
CMf80 vs GU80	0.26	0.18	0.34	< 0.001*
CM10 vs CM80	− 0.10	− 0.19	− 0.01	0.023*
CM10 vs GUf10	− 0.19	− 0.27	− 0.10	< 0.001*
CM80 vs GUf80	− 0.24	− 0.31	− 0.18	< 0.001*
GUf10 vs GUf80	− 0.16	− 0.22	− 0.09	< 0.001*
GUf10 vs GU10	0.20	0.12	0.28	< 0.001*
GUf80 vs GU80	0.24	0.16	0.31	< 0.001*
GU10 vs GU80	− 0.12	− 0.21	− 0.03	0.012*

All the comparisons between groups cured 80 s showed statistically significant lower values than groups cured 10 s, including groups CM10 and CM80 (*p* = 0.023). Similarly to initial values, groups CMf80 and GAf80 presented a lower final roughness value than all other groups. All these comparisons showed statistically significant differences between groups, except for comparisons between flowable composites: CMf10 vs GUf10 (*p* = 0.106) and CMf80 vs GUf80 (*p* = 0.403).

**Fig. 1:**
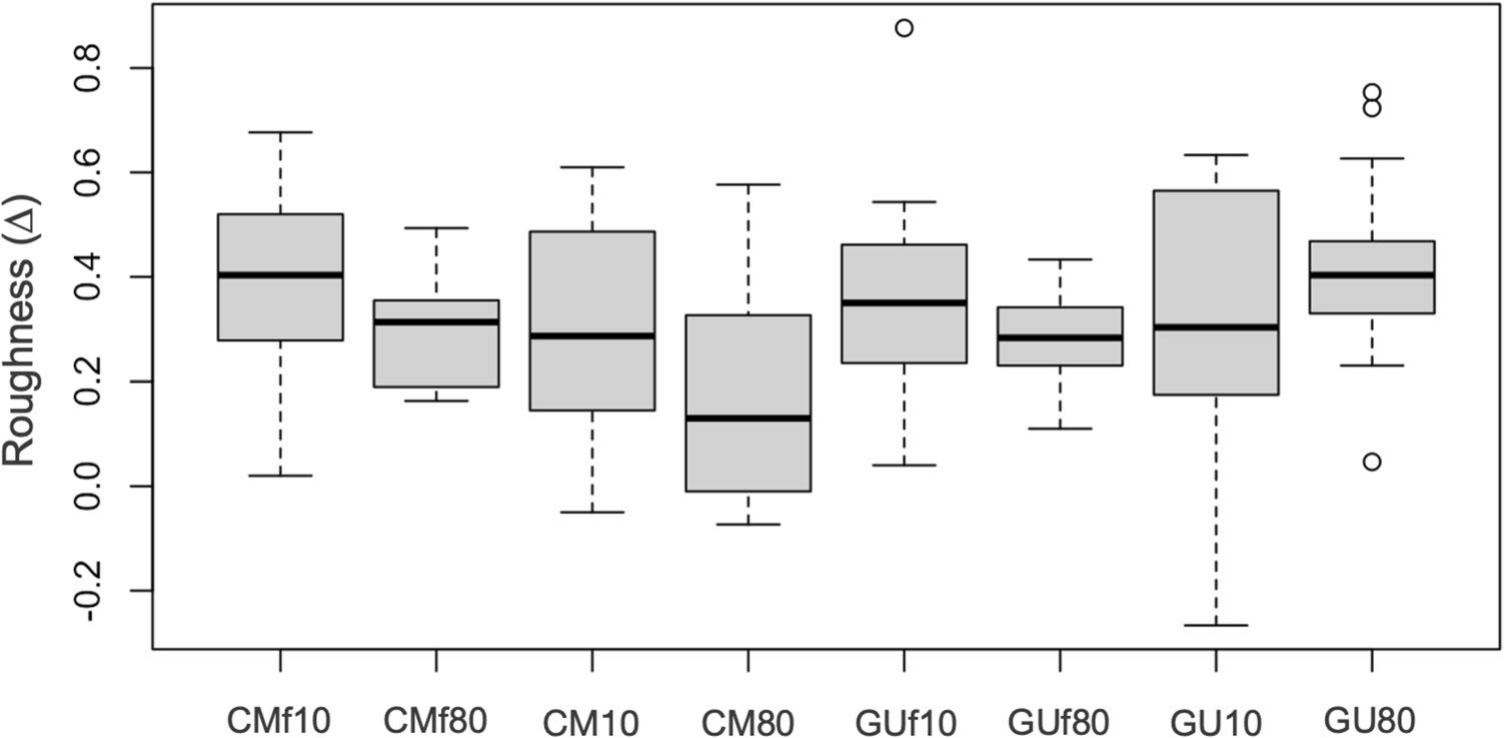
Surface roughness delta distribution through different groups

Since the initial surface roughness values between all samples were different, the delta values, i.e. the difference between final and initial roughness of each group, were compared (Fig. 1).

The two-by-two deltas comparison of the different groups revealed the presence of statistically significant differences (*p* < 0.05) (Table 5).

**Table 5:** Delta value comparison of the initial and final surface roughness between pairs of groups (* = statistically significant values)

Comparison	MD	95% Conf. Int		*p* value
CMf10 vs CMf80	− 0.102	− 0.199	− 0.006	0.038*
CMf10 vs CM10	− 0.102	− 0.225	0.022	0.103
CMf10 vs GUf10	− 0.039	− 0.157	0.079	0.507
CMf10 vs GU10	− 0.066	− 0.212	0.081	0.369
CMf80 vs CM80	− 0.127	− 0.231	− 0.022	0.019*
CMf80 vs GUf80	− 0.016	− 0.078	0.046	0.597
CMf80 vs GU80	0.120	0.031	0.208	0.010*
CM10 vs CM80	− 0.127	− 0.256	0.002	0.053
CM10 vs GUf10	0.062	− 0.060	0.185	0.308
CM80 vs GUf80	0.110	0.009	0.212	0.034*
GUf10 vs GUf80	− 0.079	− 0.172	0.013	0.091
GUf10 vs GU10	− 0.027	− 0.173	0.119	0.714
GUf80 vs GU80	0.136	0.050	0.222	0.003*
GU10 vs GU80	0.083	− 0.059	0.225	0.243

Group CMf80 presented, on average, delta roughness values 0.10 μm lower than group CMf10 (*p* = 0.038) and values 0.12 μm lower than group GU80 (*p* = 0.010).

Group CMf80 instead presented average roughness values 0.13 μm higher than those of group CM80 (*p* = 0.019). Group GUf80 presented average delta values 0.14 μm lower than those of group GU80 (*p* = 0.003), but 0.11 μm greater than those of group CM80.

Having shown lower roughness delta values, group CM80 presented better values regarding the change in surface roughness. This could mean that group CM80 composite showed a smaller increase in roughness after a simulation of 1 year of chewing cycles.

Moreover, non-statistically significant differences (*p* > 0.05) were found in the comparison between the initial and final roughness of: groups CMf10 and CM10 (*p* = 0.103), groups CMf10 and GUf10 (*p* = 0.507), groups CMf10 and GU10 (*p* = 0.369), groups CMf80 and GUf80 (*p* = 0.597), groups CM10 and CM80 (*p* = 0.053), groups CM10 and GUf10 (*p* = 0.308), groups GUf10 and GUf80 (*p* = 0.091), groups GUf10 and GU10 (*p* = 0.714), and groups GU10 and GU80 (*p* = 0.243).

### Wear

Wear values are reported in Table 6 as mean and standard deviation for each group.

**Table 6:** Final wear values for each group (mean ± Std. Dev.)

Group	Final chewing-machine values (mm)
CMf10	-0.10 (± 0.02)
CMf80	-0.06 (± 0,02)
CM10	-0.11 (± 0.02)
CM80	-0.05 (± 0.02)
GUf10	-0.13 (± 0.04)
GUf80	-0.07 (± 0.01)
GU10	-0.10 (± 0.05)
GU80	-0.06 (± 0.02)

**Fig. 2:**
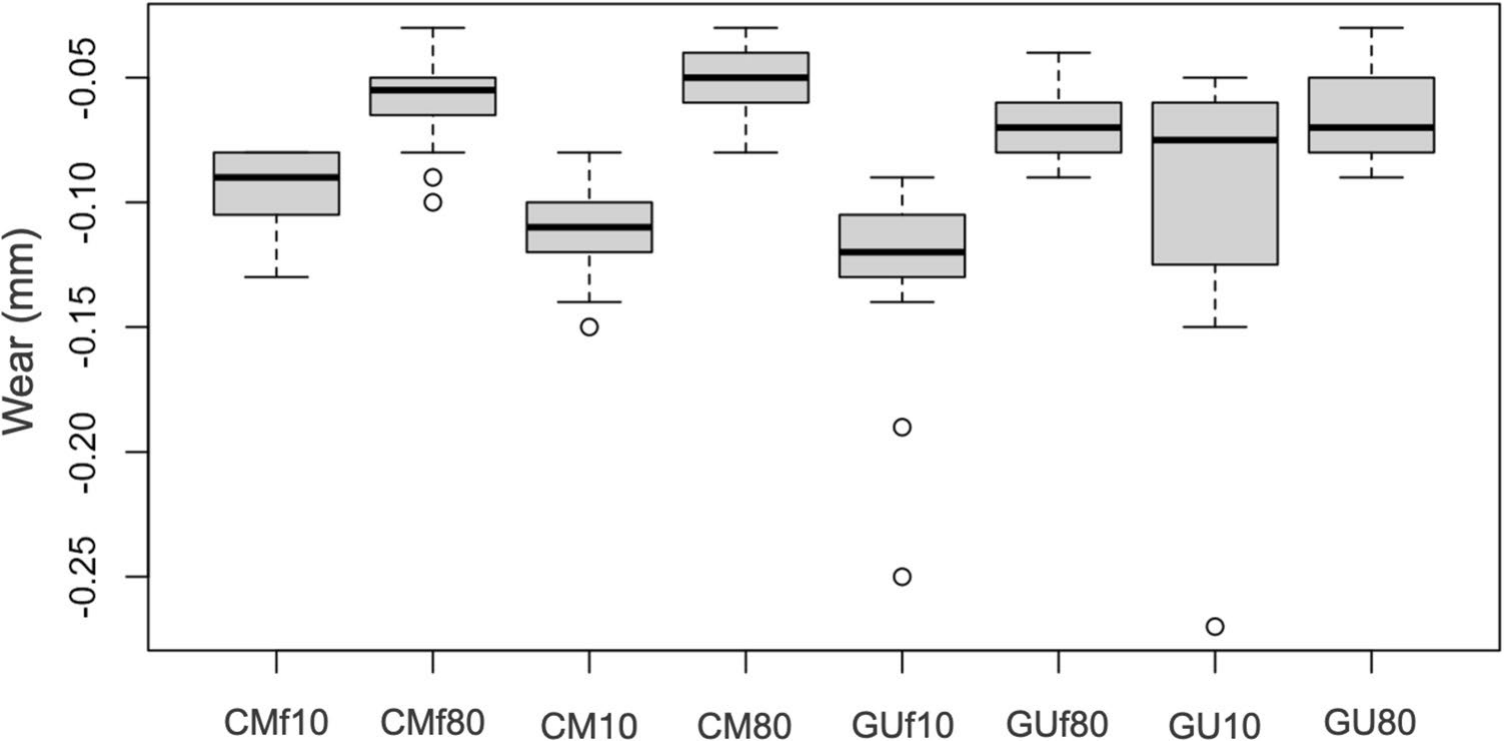
Wear value distribution through different groups

It can be observed that wear values of groups cured for 80 s were lower than the corresponding groups cured for 10 s (Fig. 2). Comparing wear values of the different groups two by two (Table 7), the presence of statistically significant differences was observed (*p* < 0.05).

**Table 7:** Wear value comparison between pairs of groups (* = statistically significant values)

Comparison	MD	95% Conf. Int		*p* value
CMf10 vs CMf80	0.038	0.027	0.048	0.000*
CMf10 vs CM10	− 0.013	− 0.024	− 0.002	0.019*
CMf10 vs GUf10	− 0.030	− 0.048	− 0.012	0.002*
CMf10 vs GU10	0.000	− 0.025	0.025	1.000
CMf80 vs CM80	0.007	− 0.004	0.017	0.230
CMf80 vs GUf80	− 0.010	− 0.020	0.001	0.077
CMf80 vs GU80	− 0.005	− 0.017	0.007	0.385
CM10 vs CM80	0.057	0.046	0.068	0.000*
CM10 vs GUf10	− 0.017	− 0.035	0.001	0.067
CM80 vs GUf80	− 0.016	− 0.026	− 0.006	0.002*
GUf10 vs GUf80	0.058	0.040	0.076	0.000*
GUf10 vs GU10	0.030	0.001	0.059	0.043*
GUf80 vs GU80	0.005	− 0.006	0.015	0.390
GU10 vs GU80	0.033	0.007	0.058	0.013*

Group CMf10 presented average wear values both lower than those of group CM10 (*p* = 0.019) and those of group GUf10 (*p* = 0.002), group GUf80 showed greater average wear values compared to those in group CM80 (*p* = 0.002), and group GUf10 showed on average wear values higher than those of group GU10 (*p* = 0.043).

Therefore, group CMf10 showed statistically significant lower wear values than both packable composites cured for 10 s, whereas group GUf10 presented significantly higher values when compared to its control.

The most effective statistically significant differences were detected between subgroups cured for 80 s and 10 s, both for packable and flowable composites. In fact, samples cured for 80 s showed always less, and therefore better, wear values.

Non-statistically significant differences (*p* > 0.05) were however found when comparing the wear of the groups: group CMf10 and GU10 (*p* = 1.000), group CMf80 and 2B (*p* = 0.230), group CMf80 and 3B (*p* = 0.077), group CMf80 and GU80 (*p* = 0.385), group CM10 and GUf10 (*p* = 0.067), and group GUf80 and GU80 (*p* = 0.390).

### SEM–FEG analysis

SEM–FEG analysis was conducted to morphologically characterize the materials sujected to chewing simulation.

The 105 × magnification images showed the wear result caused by the action of the steatite ball. The higher magnification images instead have been taken in the transition point between intact composite surface and the chewed one. In all samples, this transition area is clearly detectable.

In samples CMf10 and CMf80, the transition area between masticated and non-masticated areas is poorly detectable. Group GUf10 sample shows a transition area very well defined, whereas in the group GUf80 sample this area is less detectable and the filler particles are more present. Groups CM10 and CM80 are the ones in which the filler particles are more clarily detectable. Between all groups, GU10 and GU80 have the most evident transition area.

**Fig. 3:**
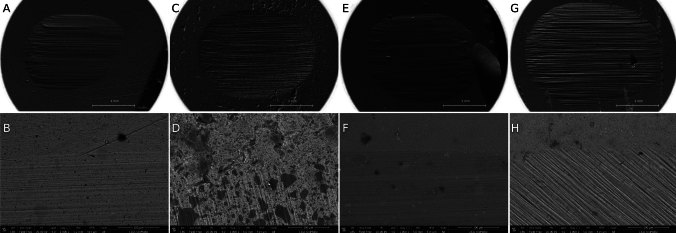
Representative FEG–SEM micrographs of the chewed surface (axial view) of the tested samples (A: sample CMf10, 105x; B: sample CMf10, 1000x; C: sample CM10, 105x; D: sample CM10, 1000x; E: sample GUf10, 105x; F: sample GUf10, 1000x; G: sample GU10, 105x; H: sample GU10, 1000x)

**Fig. 4:**
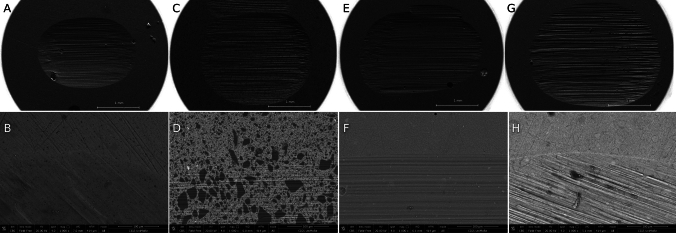
Representative FEG–SEM micrographs of the chewed surface (axial view) of the tested samples (A: sample CMf80, 105x; B: sample CMf80, 1000x; C: sample CM80, 105x; D: sample CM80, 1000x; E: sample GUf80, 105x; F: sample GUf80, 1000x; G: sample GU80, 105x; H: sample GU80, 1000x)

Figures 3 and 4 show representative SEM–FEG micrographs.

## Discussion

Surface roughness (Ra) is influenced by the filler size and percentage inside the composites. In fact, a smooth surface not only determines good esthetics, but also acceptable longevity of the restoration. On the contrary, surface irregularities lead to greater food impaction, plaque retention, gingival irritation, and secondary carious lesions [19].

Flowable composites, despite having an increased surface roughness after chewing, presenting an initial lower surface roughness, can show a lower surface roughness after mastication compared to traditional packable composites.

In a recent study, the surfaces of three flowable and one packable composites were tested, with the aim of investigating the surface quality before and after the chewing cycles (approx. 4 months). The results showed that Ra increased significantly after chewing for all composites [20]. These data seem to be compliant with the findings of the present study, because all roughness values of the masticated samples appeared to be higher than the initial ones.

In another study, it emerged that Clearfil Majesty low viscosity presented a change in surface roughness comparable to that of the control packable composites [21]. Similarly, also the results of the present study showed that Clearfill Majesty ES low viscosity presents a surface roughness comparable to traditional packable composites.

Another manuscript reported G-aenial Universal Flo as the material that presented a lower surface roughness compared to other five flowable resins tested [22].

Wear is a multifactorial process that probably cannot be adequately described with a single material property. The surfaces produced by the mastication process can provide some clues about the fatigue resistance and toughness of the composites which could be decisive in determining the effects caused by wear [23]. In vitro-simulated wear processes can provide useful information for predicting the clinical performance of composite materials [24]. Wear can be measured in volume loss (mm^3^) or in terms of maximum depth (measured in μm) [25]. In the present manuscript, wear was considered as the maximum depth obtained, and lower values were considered better indicators of clinical performance.

From the observation carried out on the images obtained by SEM analyses, it was highlighted that the transition area between the worn and intact areas of groups 1 and 3, therefore of highly loaded flow composites, appeared to be less defined. This could indicate that the mechanical stresses to which the surfaces were subjected did not undergo significant structural modifications. Groups 2 and 4, instead, presented a more evident transition area, indicating a more important structural modification, which occurred following mechanical stimuli.

Filler amounts usually vary from one composite to another and from packabkle to flowable resins. Depending on what stated by the companies and what has been published in the literature, CM and CMf composites present a 78%wt filler (and, respectively, 66% and 64% volume), whereas GU resin contains 82%wt (and 41% volume) filler and GUf 69%wt (and 50% volume). Based on the results of the present study, the authors speculate that the lower occlusal wear of CM and CMf could be attributable, in part, to their particle size. These particles are defined as, respectively, 0.7 and 0.18–3.5 µ, and they are larger than those of the other tested composites. Improved wear resistance is often related to greater filler load [26]. This trend is confimed in the present study where the percentage of filler by volume of CM and CMf is, respectively, 66% and 64%, and is higher than GU (41%) and GUf (50%).

Several authors agree on the fact that wear is influenced by the dimensions of filler particles [28, 30]. Based on the present findings however, it could be affirmed that also the filler % volume plays a crucial role in the final material wear. In fact, the more these particles are close to each other, the less space is left for the organic matrix, that is, the part where wear mostly occurs. Probably, larger particles leave less space for the organic matrix and are less distanced.

Unlike the traditional flowables, the highly filled flowables have percentage and filler particles sizes comparable to the respective traditional composites [1, 9, 15, 27, 28]. In fact, in the results of the present research, the only two comparisons that produced statistically significant differences were the CMf10 group with mean wear values 0.01 μm lower than those of the CM10 group (*p* = 0.019), and the GUf10 group with mean wear values 0.03 μm greater than those of the GU10 group (*p* = 0.043). In both cases, the comparison highlighted was that between the groups cured for 10 s, surprisingly, in the comparison between the respective groups cured for 80 s, statistically significant differences disappeared.

These results could have important implications for dental practice. While highly filled flowables have been developed as suitable for use in all kinds of direct restorations, not all these materials could have equivalent physical and mechanical features to the conventional composite. Dental practitioners should be aware of these differences when selecting materials for their direct restorations and the results of the present manuscript underscore the importance of knowledge of the physical and mechanical properties of dental materials before choosing them for daily practice [19].

Wear of four highly loaded flow composite resins were compared to a control group made of packable composite. All the tested flow composites showed higher wear values after 40,000 simulated chewing cycles than the control group. The authors concluded by suggesting that the use of flowable composite resins for small restorations should be preferable, especially in areas with low occlusal stress [27]. The results of this study highlight the fact that the tested highly filled flowable composites always present higher wear values than packable resins. These data are in contrast with those of the present study, probably due to the fact that the authors tested Clearfill Majesty packable composite that in the present article showed to be one of the best in terms of wear resistance. This aspect has also been observed in the present manuscript.

Two years later, the same study group performed wear analysis on teeth, testing four highly loaded flowable composites and a packable composite used as the control group. Wear values of highly loaded flowable composites were comparable to those of the packable composite, showing that good wear resistance is strictly dependent of the percentage and size of the filler inside the resin, and therefore flowable composites with greater amounts of filler (respectively, 81%wt, 71%wt, 67%wt, 67%wt) had similar wear values to the control group (78%wt) [1]. This paper showed that the wear values of flowable composites are dependent on the size of the filler particles, and the results related to higly loaded flowable composites are similar to those of the present study that used the same CMf and CM resins. Similar conclusions were obtained in other studies that evaluated wear on several composite resins [26, 29].

Further, a recent study compared the wear of four flowable bulk resins with four flowable resins after chewing simulation of 400,000 cycles. In terms of both volume loss (mm^3^) and maximum depth (μm), G-aenial Bulk Injectable and G-aenial Universal Flow had much lower values than the other tested resins. For this reason, among the conclusions of the study, it is stated that these resins seem to be indicated for restorations of the posterior sectors in occlusal contact areas [28]. Similarly to the results of the present study, the paper by Ujiie et al. affirm that wear values of highly loaded flowables are lower or similar to those of packable composites. Highlighting the fact that GU and GUf composites present a similar wt percentage (respectively, 82 and 69%), it can be ascertained that both materials have the features to be used in occlusal areas.

A recent in vitro study aimed to comparatively assess the wear resistance of three conventional and three flowable composites containing different filler types using thermomechanical chewing simulation. Specimens were subjected to wear using a thermocycler chewing simulator against 6-mm diameter steatite balls for 240,000 cycles. The digital profiles of the treated sample surfaces were scanned using a laser scanner, and the volume loss and maximum depth of loss were calculated. The wear volume loss and loss depth of nanofilled composites were significantly higher than those of the other composite filler types, with no significant difference in either parameter between the nanohybrid and submicron-filled composite groups. With respect to apparent viscosity, wear volume loss and depth loss of conventional composites were significantly lower than those of the flowable composites. The type of composite filler and its viscosity significantly influence the in vitro wear resistance of the material [9]. Similarly to the present study, the authors affirm that the amount of wear increases when the composite filler percentage decreases.

Another recent in vitro study aimed to evaluate and compare the roughness and hardness of two bulk-fill flowable composites, two conventional flowable composites and one high-strength universal injectable composite. Flowable bulk-fill composites and high-strength injectable composite showed similar results in terms of hardness, which appeared to be statistically higher when compared with both traditional flowable composites hardness. Flowable bulk-fill composites showed significantly higher roughness values than both traditional flowable composites and high-strength injectable composite [15].

By several studies in literature, it seems evident that not only the filler properties and the polymer matrix, but also the time taken for photopolymerization affects the mechanical properties of the composite [30, 31]. Greater polymerization increases the mechanical properties of resistance to wear and fracture and makes the color of the composite more stable over time [17, 18].

In a recent study, three bulk nanohybrid composites and a control group were polymerized for 10 s or 80 s and tested for bacterial adhesion and surface roughness. Results showed that samples cured for 80 s presented lower surface roughness compared to samples cured for 10 s [32]. In agreement with literature data, the results of the present experiment showed that, regarding both the initial and final surface roughness, there were evident differences between the subgroups cured for 80 s compared to those cured for 10 s. Despite this, in terms of delta, and therefore in terms of difference between the initial and final roughness, no statistically significant differences were found, except for that in Clearfil Majesty ES Flow composite, in which the comparison between group CMf10 and CMf80 was the only one to highlight a significant difference: 0.1 μm (*p* = 0.038). In terms of wear, the results clearly showed that for each group, there were statistically significant differences between the subgroups cured for 80 s and those cured for 10 s. In fact, group CMf80 presented average wear values lower than 0.04 μm compared to those in group CMf10 (*p* = 0.000): both in the comparison between group CM80 and CM10, and in the comparison between group GUf80 and GUf10, there were differences in wear on average of 0.05 μm (*p* = 0.000) and also in the comparison between the GU80 and GU10 group, there was a difference in wear of 0.03 μm (*p* = 0.013) again in favor of the 80 s polymerized group. Thus, samples cured for 80 s had a statistically lower wear value for each of the composites tested.

Polymerization shrinkage stress of resin composite materials may have a negative impact on the clinical performance of bonded restorations. Shrinkage stress development has to be considered a multi-factorial phenomenon, and several restorative techniques aiming at stress reduction seem to have limited applicability, since their efficiency varies depending upon the materials used. Since the understanding of this matter has remarkably increased, the development of new restorative techniques and materials may help minimize this problem [33, 34].

It is mandatory to highlight that the present research has been conducted in vitro and that the interpretation of the results could present some limitations. While in vitro studies provide valuable insights, they may not fully replicate the complex environment of the oral cavity and might not perfectly replicate actual masticatory forces and conditions over extended periods. Additionally, no thermocycling was considered in the present research. Composites aging in the oral cavity and tooth brushing could modify composites’ roughness and wear.

The null hypotheses are confirmed with regard to surface roughness and wear. In fact, it has been demonstrated that there are no differences between highly filled flowable composites and traditional packable composites, when the same polymerization time was used. The last null hypothesis must be instead rejected, since differences in surface roughness and wear were found between 10 s polymerized composites and 80 s polymerized ones.

Further research, particularly in vivo and with a broader range of materials, should replicate the actual clinical condition through long-term clinical trials and would enhance the robustness and applicability of its findings. However, the value of in vitro research should not be undermined, since it enables the investigation of one or several “isolated” factors on the mechanical properties of dental materials, without the inevitable variability between subjects in a clinical study.

## Conclusions

After chewing simulation, the higlh filler content resin flows exhibited surface roughness and wear comparable to their paste counterparts. Increasing the curing time resulted in improved mechanical performances of all materials tested.

## Data Availability

Data are available from the corresponding author.
